# Retinoprotective Effects of TAT-Bound Vasoactive Intestinal Peptide and Pituitary Adenylate Cyclase Activating Polypeptide

**DOI:** 10.1007/s12031-018-1229-5

**Published:** 2018-12-12

**Authors:** Tamas Atlasz, D. Werling, S. Song, E. Szabo, A. Vaczy, P. Kovari, A. Tamas, D. Reglodi, Rongjie Yu

**Affiliations:** 1 Department of Anatomy, Medical School, MTA-PTE PACAP Research Group, University of Pecs, Pecs, Hungary; 2 Department of Sportbiology, University of Pecs, Pecs, Hungary; 3 Janos Szentagothai Research Center, University of Pecs, Pecs, Hungary; 4 Institute of Biomedicine, Jinan University, Guangzhou, China

**Keywords:** Eye drops, TAT, Bio-barriers, Retinal protection

## Abstract

Vasoactive intestinal peptide (VIP) and pituitary adenylate cyclase activating polypeptide (PACAP) belong to the same peptide family and exert a variety of biological functions. Both PACAP and VIP have protective effects in several tissues. While PACAP is known to be a stronger retinoprotective peptide, VIP has very potent anti-inflammatory effects. The need for a non-invasive therapeutic approach has emerged and PACAP has been shown to be retinoprotective when administered in the form of eye drops as well. The cell penetrating peptide TAT is composed of 11 amino acids and tagging of TAT at the C-terminus of neuropeptides PACAP/VIP can enhance the traversing ability of the peptides through the biological barriers. We hypothesized that TAT-bound PACAP and VIP could be more effective in exerting retinoprotective effects when given in eye drops, by increasing the traversing efficacy and enhancing the activation of the PAC1 receptor. Rats were subjected to bilateral carotid artery occlusion (BCCAO), and retinas were processed for histological analysis 14 days later. The efficiency of the TAT-bound peptides to reach the retina was assessed as well as their cAMP increasing ability. Our present study provides evidence, for the first time, that topically administered PACAP and VIP derivatives (PACAP-TAT and VIP-TAT) attenuate ischemic retinal degeneration via the PAC1 receptor presumably due to a multifactorial protective mechanism.

## Introduction

Vasoactive intestinal peptide (VIP) and pituitary adenylate cyclase activating polypeptide (PACAP) belong to the same peptide family. PACAP exists in 27 and 38 amino acid forms, and the shorter peptide shows 67% homology with VIP. They also share their receptors: VPAC1 and VPAC2 receptors bind both VIP and PACAP. However, PACAP also has a specific PAC1 receptor, which only binds PACAP. PACAP has a widespread occurrence in the body and a broad array of functions (Reglodi and Tamas 2016). Among others, PACAP influences gastrointestinal, urinary and cardiovascular functions (Heppner et al. 2018; Parsons and May 2018; Reglodi et al. 2018a), plays a role in reproduction and pregnancy (Lajko et al. 2018; Reglodi et al. 2012; Ross et al. 2018), has diverse behavioral and cognitive functions (Farkas et al. 2017; Gupta et al. 2018; Han et al. 2014; King et al. 2017), plays roles during both early development and aging (Fulop et al. 2018; Reglodi et al. 2018b; Watanabe et al. 2007), as well as influences the functions of both endocrine and exocrine glands (Bardosi et al. 2016; Egri et al. 2016; Prevost et al. 2013; Sasaki et al. 2017). VIP has also been shown to have diverse actions in addition to the originally described vasodilatory effects (Gozes 2008; Hill et al. 2007; Moody and Gozes 2007; Vu et al. 2015). VIP was originally isolated as a vasoactive peptide in the airways, later confirmed in the gastrointestinal tract (Vu et al. 2015). VIP is involved, among others, in immunomodulatory pathways (Abad and Tan 2018; Carrión et al. 2016; Jimeno et al. 2014), in nervous system development and in the acquisition of certain neurological disorders (Maugeri et al. 2018a, b; Morell et al. 2012).

Both PACAP and VIP exert protective effects in several tissues (Brifault et al. 2016; Giladi et al. 2007; Reglodi et al. 2011; Shioda and Gozes 2011). VIP has stronger anti-inflammatory effects (Olson et al. 2015), while PACAP is a more potent antiapoptotic peptide (Reglodi et al. 2018c). In the eye, VIP and PACAP have various biological effects. Among others, PACAP has been described to participate in the iris sphincter functions (Yoshitomi et al. 2002), stimulates tear secretion (Nakamachi et al. 2016; Shioda et al. 2018) and modulates its composition (Gaal et al. 2008), influences corneal keratinization and wound repair (Ma et al. 2015; Nakamachi et al. 2016) and is involved in the sensory innervation of the ocular surface (Wang et al. 1996). PACAP and VIP also have protective effects on the corneal endothelium (Koh et al. 2017; Maugeri et al. 2018a, b). Both peptides and their receptors are distributed also in the retina, where they are involved in information processing of visual stimuli (Akrouh and Kerschensteiner 2015; Atlasz et al. 2016; Dragich et al. 2010; Pérez de Sevilla Müller et al. 2017; Webb et al. 2013) and have trophic functions (Endo et al. 2011; Fabian et al. 2012).

The retinoprotective effects of PACAP are well-documented and have been proven in different injury models, such as excitotoxic, ischemic, UV light-induced, traumatic, diabetic and oxygen-induced injuries (Atlasz et al. 2008, 2011, 2016; Kvarik et al. 2016; Shioda et al. 2016; Szabadfi et al. 2016; Vaczy et al. 2016). VIP, on the other hand, seems to be a less potent retinoprotective peptide. VIP has been shown to exert retinoprotective effects mainly in conditions involving inflammatory processes (Shi et al. 2016; Tunçel et al. 1996). However, in ischemic retinopathy, VIP was proven to be ten times less active than PACAP (Szabadfi et al. 2012). In most in vivo retinal disease models, PACAP and VIP have been administered as intravitreal injection in order to guarantee that the injected peptides reach the retina in high enough concentrations to exert protective effects. As PACAP exerts dramatic retinoprotective effects proven by dozens of studies, therapeutic use is implied and so the need for a non-invasive approach has emerged. One possible approach is to enhance cell penetration of these peptides. The cell penetrating peptide TAT (GRKKRRQRRRPQ) is derived from the HIV Tat protein (Schwarze et al. 1999). TAT has protein transduction domains (PTDs) with the ability to efficiently traverse cellular membranes. TAT can not only transfer different types of molecules (peptides, large molecular proteins, DNAs) into a variety of cell types, but can also bring the linked molecules across many biological barriers such as the blood-brain barrier (BBB), mucosal barrier and lung respiratory epithelium in vivo (Dietz and Bähr 2004). Our previous study reported that the tagging of TAT at the C-terminus of neuropeptides PACAP/VIP enhanced the traversing ability of the peptides through the biological barriers, such as BBB and blood–air barrier and blood–testis barrier (Yu et al. 2012a, b). Furthermore, we found that VIP-TAT has higher activity on the activation of PACAP preferring PAC1receptor than VIP (Yu et al. 2014). The structure analysis showed that TAT has a two-dimensional structure similar to that of PACAP(28–38), and PACAP(28–38) has been shown to facilitate the binding and the activation of PAC1-R (Vaudry et al. 2009).

PACAP in the form of eye drops has first been shown to exert local effects on the cornea. It has been shown to enhance corneal wound regeneration and nerve regrowth after injuries (Fukiage et al. 2007; Ma et al. 2015; Nakamachi et al. 2016; Shioda et al. 2018). Similarly, VIP has been shown to enhance corneal wound repair after alkali burn injury (Tuncel et al. 2016). Our recent studies have demonstrated that PACAP eye drops not only lead to topical effects, but PACAP is able to pass the ocular barriers and reach the retina, where it can exert retinoprotective effects (Werling et al. 2016, 2017). We hypothesize that the passage through ocular layers can be further enhanced by the binding of TAT peptide, which is known to increase passage of peptides through biological barriers. We have previously shown that intravitreally administered VIP is able to protect the retina against hypoperfusion-induced injury, but only in a dose ten times higher than that of PACAP (Szabadfi et al. 2012). We hypothesized that TAT-bound PACAP and VIP (PACAP-TAT, VIP-TAT) could be more effective in exerting retinoprotective effects when given in eye drops, by increasing the traversing efficacy and enhancing the activation of the PAC1 receptor. The aim of the present study, therefore, was to investigate the potential retinoprotective effects of PACAP-TAT and VIP-TAT administered in eye drops following bilateral carotid artery occlusion (BCCAO)-induced retinopathy in rats.

## Materials and Methods

### Materials

The peptides PACAP38, VIP, PACAP-TAT (tagging TAT at the C-terminus of PACAP38) and VIP-TAT (tagging TAT at the C-terminus of VIP) were chemically synthesized by GL Biochem Ltd. (Shanghai, China).

### Peptides Labeled with Fluorescein Isothiocyanate

In order to trace the drugs, peptides were labeled with fluorescein isothiocyanate (FITC) using a FITC Protein Labeling Kit from ChangRu Biotech Ltd. (Guangzhou, China) according to the manufacturer’s protocol. After the labeling reaction, gel filtration was used to remove the free FITC. In order to determine the amount of the residual free FITC, the peptides were submitted to ultrafiltration using Amicon Ultra – 0.5 mL (Millipore, USA) with a molecular sieve of 2000 Da. After centrifugation (1000×*g*, 10 min) the peptide was subjected to fluorescence measurements performed with the multi-wavelength scanner Victor 3 (GE, USA) at the excitation of 495 nm and the emission of 520 nm. Protein concentrations were determined using the K4000 Bradford Protein Quantification Kit (Innovative, Guangzhou, China). The labeling efficiency was calculated using the following formula: label efficiency (LE) = fluorescence value (FV)/peptide mass (mol) (PM), representing the fluorescence intensity (AU) per mol of peptide (mol).

### The Efficiency of Reaching the Retina

Male rats with body weight from 160 to 180 g were purchased from the Medical and Experimental Animal Center (Guangdong, China). Rats were randomly assigned to one of the experimental groups (7 rats per group) and subjected to eye drops with FITC labeled peptides (100 nmol/kg) and PBS as control. Rats were sacrificed by anesthesia 2 h after the eye drop administration and the retina was separated, weighed, washed three times with PBS and divided into two parts. One part was prepared on the glass slide with glycerin and subjected to fluorescence microscopic observation of FITC with 495 nm excitation/520 nm emission. All images, focused on the left upper regions of the retina, were taken with 500 ms exposure time. The other part of the retina was subjected to grinding and ultrasonication in PBS at a concentration of 100 mg weight tissue per milliliter of PBS. The supernatant was collected by centrifugation and the fluorescence intensity in the supernatant (100 uL) was determined. The valid fluorescence intensity (FI) for each sample treated with FITC labeled peptide was corrected by subtracting the fluorescence value of the sample treated with PBS, which was used as a blank background. The Efficiency of Traversing Eye to Retina (EtE) was expressed as the percentages of the FITC labeled peptide mass in the retina to the total FITC labeled peptide mass. The EtE was calculated using the following formula: EtE = tFI/LE/PW × 100% (tFI presents the fluorescence intensity of retina (arbitration unit, AU); LE presents the label efficiency of each peptide which has been determined above; PW presents the peptide mass (mole)).

### cAMP Accumulation Assay

PAC1-CHO cells cultured in Dulbecco’s Modified Eagle’s Medium (DMEM) at 37 °C were scraped off the surface with rubber policeman, washed twice with PBS and the density of the cells was adjusted to 2 × 10^6^ /mL. Peptides were added to 500 uL cells suspension with the corresponding varying working concentrations of the detected factor. After incubation at 37 °C for 5-10 min cells were harvested and the lysates were subjected to cAMP quantification using the enzyme immunoassay kit for cAMP (Biyuntian, Shanghai, China), following the manufacturer’s instructions. Protein concentration of each sample was determined using BCA assay, and the cAMP level of each sample was calculated following the formula: cAMP level (pmol/mg protein) = cAMP concentration (pmol/mL)/protein concentration (mg/mL). The cAMP level in each sample was plotted as the percentage (%) of the maximal cAMP level in cells treated with PACAP27 versus the logarithmic value of the peptide concentrations. All experiments were run with at least four parallel samples and were repeated three times.

### Histological Procedure in the Retina

Adult male rat litters were housed in the animal facility in individual cages in a 12 h light-dark cycle with food and water ad libitum. Animal housing, care and application of experimental procedures were in accordance with institutional guidelines under approved protocols (No: BA02/2000–26/2017, University of Pecs). Under isoflurane anesthesia, common carotid arteries were exposed on both sides through a midline incision and then ligated with a 3–0 filament. A group of animals (sham group) underwent all steps of the operating procedure except ligation of the carotid arteries. Immediately following the operation, the right eye of the animals was treated with derivatives of PACAP (PACAP-TAT /*n* = 17/ or VIP-TAT /*n* = 17/) eye drops (1 μg/drop). Dose and schedule of the eye drop treatments were based on our previous experiments (Werling et al. 2016). In the experiment for histological analysis, the different derivatives were dissolved in benzalkonium solution for ophthalmic use (solutio ophthalmica cum benzalkonio (SOCB)). The left eye, serving as a control, was treated with vehicle containing neither PACAP-TAT nor VIP-TAT. Animals were treated for five consecutive days, twice a day with one drop of drug, under brief isoflurane anesthesia (max. 5 min).

Fourteen days after the operation, rats (*n* = 10 SHAM and *n* = 24 BCCAO) were killed with anesthetic and the eyes were processed for histology. The eyes were removed and the retinas were solved in phosphate buffered saline (PBS), fixed in 4% paraformaldehyde dissolved in 0.1 M phosphate buffer (PB) (Sigma, Budapest, Hungary) and embedded in Durcupan ACM resin (Sigma, Budapest, Hungary). Retinas were cut at 2 μm and stained with toluidine blue dye (Sigma, Hungary). Sections were mounted in DPX medium (Sigma, Hungary) and photographs were taken with a digital CCD camera using the Spot program. Central retinal areas within 1 mm from the optic nerve were used (*n* = 5 measurements from one tissue block). The following parameters were measured: (i) cross-section of the retina from the outer limiting membrane (OLM) to the inner limiting membrane (ILM), (ii) the width of individual retinal layers (outer nuclear layer [ONL], outer plexiform layer [OPL], inner nuclear layer [INL], inner plexiform layer [IPL]), (iii) the number of cells/100 μm section length in the GCL, and the (iv) number of cells/1 μm2 in the OPL and in the IPL. Results are presented as mean ± SEM. Statistical comparisons were made using the two-way ANOVA test followed by Tukey’s post hoc analysis.

## Results

### TAT Tagging Enhances the Efficiency of Reaching Retina

**Fig. 1:**
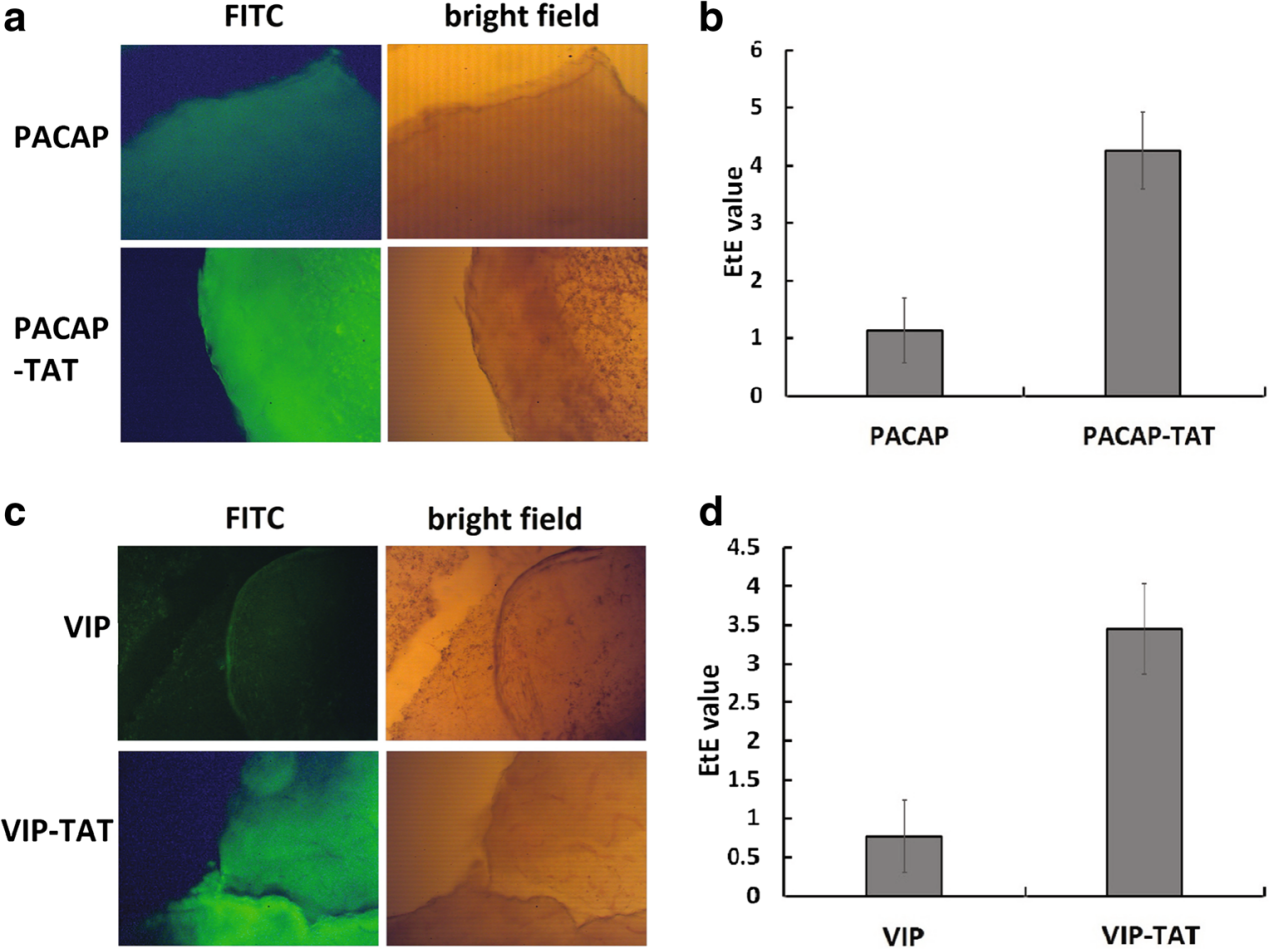
The efficiency of FITC labeled PACAP/PACAP-TAT (**a**, **b**) and VIP/VIP-TAT (**c**, **d**) traversing to retina given in eye drops. The retina was separated 2 h after the eye drops and submitted to the fluorescence microscopic observation of FITC fluorescence signal **(A, C)** and the calculation of Efficiency of Traversing Eye to Retina (EtE) (**b**, **d**). The data are means ± SEM of four experiments

The fluorescence imaging results of the retina after the treatment with eye drops of FITC labeled peptides (Fig. 1) showed that the FITC fluorescence density per area unit in the retina treated with eye drops of PACAP-TAT (Fig. 1a) and VIP-TAT (Fig. 1c) was much higher than in retinas treated with eye drops of PACAP/VIP, indicating that PACAP-TAT/VIP-TAT reached the retina more efficiently than PACAP/VIP. The calculation of the Efficiency for Traversing Eye to Retina (EtE) showed that the PACAP-TAT/VIP-TAT reached the retina with the efficiency (3.66 ± 0.67%, 3.05 ± 0.58%) about three-fold that of PACAP/VIP (1.23 ± 0.56%, 0.97 ± 0.47%), respectively.

### TAT-Tagging Enhanced the Activity of PACAP/VIP on the Activation of PAC1-R

**Fig. 2:**
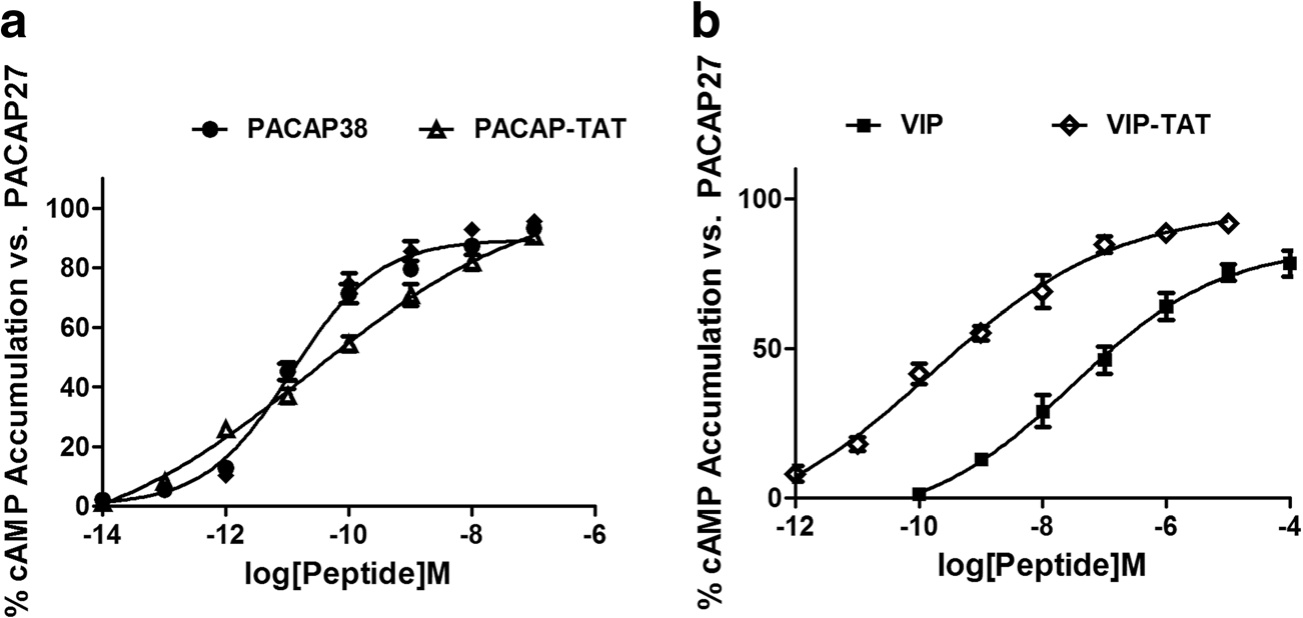
cAMP assay results showing the effects of PACAP/PACAP-TAT (**a**) and VIP/VIP-TAT (**b**) on the activation of PAC1-R. The intracellular cAMP accumulation in PAC1-CHO cells induced by PACAP38(), PACAP-TAT(), VIP() and VIP-TAT () in their respective effective working concentration was plotted as the percentage (%) of the maximum cAMP level induced by PACAP27. The data are means ± SEM of four experiments

The results of cAMP assay (Fig. 2) showed that PACAP-TAT had EC50 of 23.6 ± 4.4 pM significantly higher than PACAP38 with 11.7 ± 3.1 pM, whereas VIP-TAT had EC50 of 0.14 ± 0.02 nM about 1/200 of the EC50 of VIP 30.1 ± 4.1 nM. These results showed that TAT-tagging enhanced the activity of VIP on the activation of PAC1-R, but inhibited the activity of PACAP38.

### Morphological Analysis in the Retina after BCCAO

**Fig. 3:**
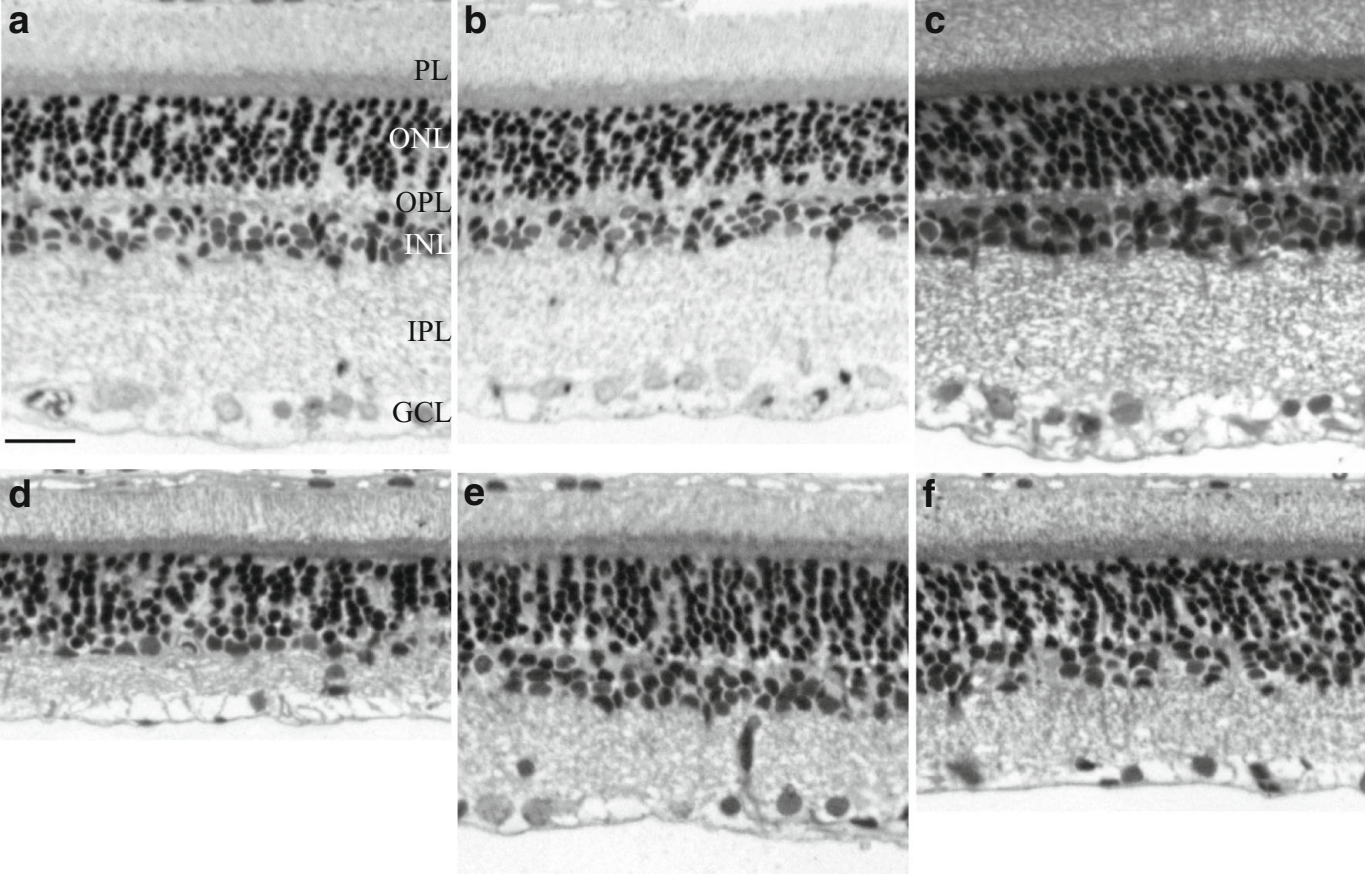
Light microphotographs of retinal sections. Retinal tissue from BCCAO+SOCB (**d**) showed severe degeneration compared to SHAM+SOCB (**a**), SHAM + PACAP-TAT (**b**) or SHAM + VIP-TAT (**c**). The retinal layers of BCCAO+SOCB rats following treatment with eye drops containing PACAP-TAT (**e**) or VIP-TAT (**f**) showed only mild degeneration. Abbreviations: PL, photoreceptor layer; ONL, outer nuclear layer; OPL, outer plexiform layer; INL, inner nuclear layer; IPL, inner plexiform layer; GCL, ganglion cell layer. Scale bar: 20 μm

**Fig. 4:**
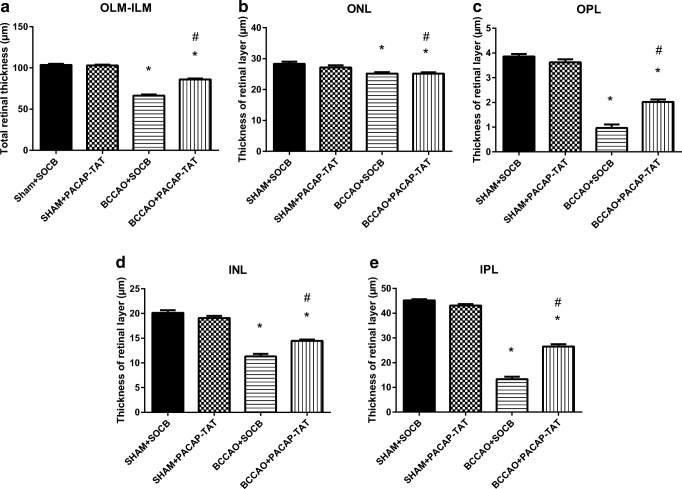
Quantification of retinal layers in SHAM+SOCB, in SHAM+PACAP-TAT, in BCCAO+SOCB, and in BCCAO+PACAP-TAT animals; the right eye was treated with PACAP-TAT eye drops, the left eye served as controls receiving only SOCB. Comparison of all retinal layers (**a–e**). Morphometric analysis showed that treatment with PACAP-TAT eye drops improved the structure of all the retinal layers. Statistical significance (*p < 0.05 vs. SHAM+SOCB retinas, #*p* < 0.05 vs. BCCAO+SOCB retinas) was calculated by two-way ANOVA followed by Fischer’s post hoc test

**Fig. 5:**
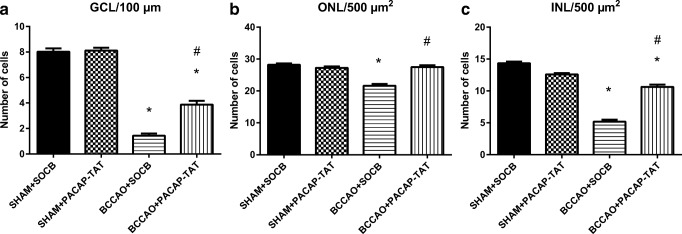
Quantification of the number of cells/100 μm GCL length (**a**), the number of cells/500 μm^2^ ONL (**b**) and INL (**c**) areas in SHAM+SOCB, in SHAM+PACAP-TAT, in BCCAO+SOCB, and in BCCAO+PACAP-TAT animals. Statistical significance (*p < 0.05 vs. SHAM+SOCB retinas, #*p* < 0.05 vs. BCCAO+SOCB retinas) was calculated by two-way ANOVA followed by Bonferroni’s post hoc test

**Fig. 6:**
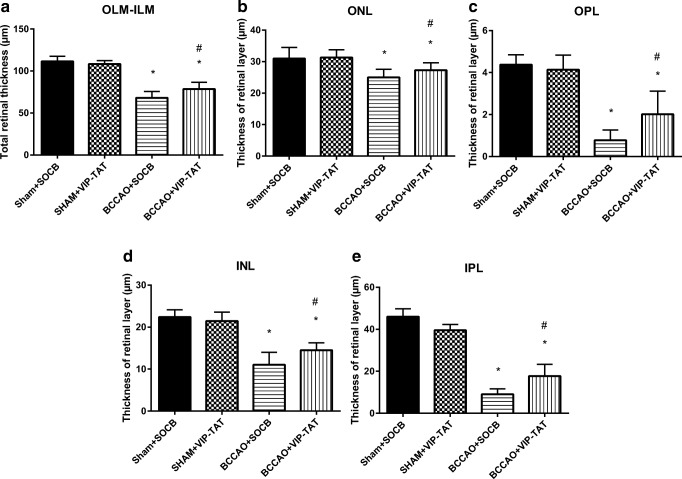
Quantification of retinal layers in SHAM+SOCB, in SHAM+VIP-TAT, in BCCAO+SOCB, and in BCCAO+VIP-TAT animals; the right eye was treated with VIP-TAT eye drops, the left eye served as control receiving only SOCB. Comparison of all retinal layers (**a–e**). Morphometric analysis showed that treatment with VIP-TAT eye drops improved the structure of all the retinal layers. Statistical significance (*p < 0.05 vs. SHAM+SOCB retinas, #*p* < 0.05 vs. BCCAO+SOCB retinas) was calculated by two-way ANOVA followed by Fischer’s post hoc test

**Fig. 7:**
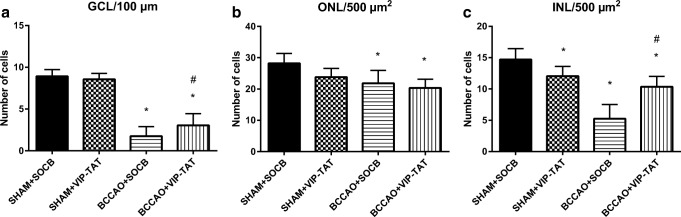
Quantification of the number of cells/100 μm GCL length (**a**), the number of cells/500 μm^2^ ONL (**b**) and INL (**c**) areas in SHAM+SOCB, in SHAM+VIP-TAT, in BCCAO+SOCB, and in BCCAO+VIP-TAT animals. Statistical significance (*p < 0.05 vs. SHAM+SOCB retinas, #*p* < 0.05 vs. BCCAO+SOCB retinas) was calculated by two-way ANOVA followed by Bonferroni’s post hoc test

Carotid occlusion caused significant thickness reduction in all layers compared to sham animals. The most marked reduction in thickness was found in the outer and inner plexiform layers, and as a consequence, the total retinal thickness (OLM-ILM) was significantly less than in control retinas (Figs. 3 and 4). PACAP derivatives (PACAP-TAT, VIP-TAT) administration alone in sham animals did not cause any changes in the retinal thickness (Figs. 3 and 4). Eye drops containing PACAP-TAT or VIP-TAT caused significant amelioration in all retinal layers compared to the sham group. The thickness of the major retinal layers was significantly larger than that of the degenerated ones (Figs. 3 and 4). This was especially conspicuous in the OPL, which almost disappeared in several BCCAO-induced degenerated retinas and was preserved in PACAP-TAT or VIP-TAT-treated animals. The number of cells in different retinal layers also changed. BCCAO led to a significant cell loss in the ONL, INL and GCL. Eye drops with PACAP-TAT counteracted the effects of the BCCAO in all nuclear layers. The cell numbers in the GLC/100 μm, in the ONL/500 μm2 and in the INL/500 μm2 were significantly higher compared to the BCCAO-induced degenerated retinas. VIP-TAT administration also led to reduced cell loss in almost all nuclear layers, except in the ONL/500 μm2 (Figs. 5, 6 and 7).

## Discussion

In the present study we demonstrated the efficacy of TAT-bound PACAP and VIP peptides to reach the retina and exert a retinoprotective effect in a model of ischemic retinopathy in rats. The retinoprotective effects of PACAP are well-documented in models of many different retinopathies (Atlasz et al. 2011, 2016; Shioda et al. 2016). Intravitreal injections of PACAP have been shown to lead to robust retinoprotective effects in various models of retinal injuries (Atlasz et al. 2016). The protective effects have been demonstrated to affect all neuronal cell types, from ganglion cells (Atlasz et al. 2010; Shoge et al. 1999) to photoreceptors and bipolar neurons (Szabadfi et al. 2016), the two main interneuronal types, amacrine and horizontal cells (Szabadfi et al. 2012) and the main glial cells, Muller glial cells (Nakatani et al. 2006; Werling et al. 2016). Furthermore, PACAP is an endogenous regulator of retinal microglial cells/macrophages, important in certain pathological conditions (Wada et al. 2013). PACAP not only affects the neurons and glial cells of the retina leading to retinoprotection, but also helps to preserve the integrity of the blood-retinal barrier (Scuderi et al. 2013) and protects the retinal pigment epithelial cells against oxidative stress injury, a process important in preservation of the outer barrier of the retina (Fabian et al. 2012). Furthermore, PACAP influences retinal vasculogenesis, especially under pathological conditions (Kvarik et al. 2016).

VIP has also been shown to have effects in the visual system according to some studies, although most results point to its involvement in photic neuronal transmission rather than its trophic effects (Akrouh and Kerschensteiner 2015; Dragich et al. 2010; Pérez de Sevilla Müller et al. 2017; Webb et al. 2013). VIP is an important neuromodulator along the visual transmission pathways, not only in the retina, but all the way to the cortex where it influences visual information processing (Galletti and Fattori 2018; Wilson and Glickfeld 2014). Regarding retinoprotection, a few studies indicate that VIP may also exert trophic effects in certain retinal injuries. Among others, VIP has been shown to protect retinal ganglion cells against excitotoxic injury in vitro (Shoge et al. 1998). VIP also protected against ischemia-reperfusion injury induced by ophthalmic vessel ligation (Tunçel et al. 1996), where both systemic and intravitreal VIP decreased oxidative stress as shown by reduced malondialdehyde levels. This led to a more preserved histological structure, which is in accordance with our present findings. Our earlier study, using the same hypoperfusion model used in the present study, showed that intravitreal VIP administration led to retinal morphological amelioration, but only at doses ten times higher than PACAP (Szabadfi et al. 2012). In the present study, we show a similar degree of protection, using TAT-bound VIP. VIP’s actions include not only direct effects, but also indirect effects, through stimulation of activity-dependent neurotrophic protein (ADNP) and its short fragment NAP, with highly potent neuroprotective effects. Both ADNP and NAP exerted strong protection against a variety of stress factors (Steingart et al. 2000). In the retina, NAP protected against laser-induced retinal damage (Belokopytov et al. 2011), to decrease hypoxia-inducible factor levels in a model of diabetic retinopathy (D’Amico et al. 2017, 2018; Maugeri et al. 2017), to prevent apoptotic cell death (Scuderi et al. 2014) and to promote neuronal growth after hypoxia-induced injury (Zheng et al. 2010). VIP also affects autonomic reflexes and choroidal blood flow, which eventually affects retinal blood supply (Bill and Sperber 1990). Applying VIP on the ocular surface in the form of eye drops has so far been shown to exert local effects on the cornea.

Regarding ischemic injury, PACAP has been shown to be protective in most cell layers affected in BCCAO-induced retinal ischemia. VIP was previously proven to be ten times less effective: intravitreal 100 pmol VIP, in contrast to the same dose of PACAP, led to no ameliorating effect on the retinal structure. However, 1000 pmol intravitreal VIP produced a protective effect. As eye drops, VIP was not effective alone (not shown). However, in our present study, we confirm that VIP bound to TAT peptide could effectively traverse the ocular barriers and exert a neuroprotective effect in the retina. PACAP-TAT did not prove to have significantly higher retinoprotective efficacy than untagged PACAP, but VIP exerted much stronger retinoprotective effects when bound to TAT. These results were consistent with our previous report that TAT with similar structure with PACAP(28–38) endowed VIP with higher affinity for PAC1-R (Yu et al. 2014). As for PACAP38, the tagging with TAT at the C-terminus of PACAP38 would be redundant and interfere with the receptor binding. This may be the reason why TAT tagging had some negative effect on PACAP38’s activity on the activation of PAC1-R. Also, as VIP has been implicated in a variety of other ocular diseases as a possible therapeutic approach (Berger et al. 2010; Cakmak et al. 2017; Satitpitakul et al. 2018), our results with topical applications leading to retinoprotection may open new therapeutic approaches.

In summary, our present study provides evidence, for the first time, that topical administration of PACAP and VIP derivatives (PACAP-TAT and VIP-TAT) dissolved in SOCB attenuate ischemic retinal degeneration via the PAC1 receptor presumably due to a multifactorial protective mechanism.
